# Prognostic biomarker SMARCC1 and its association with immune infiltrates in hepatocellular carcinoma

**DOI:** 10.1186/s12935-021-02413-w

**Published:** 2021-12-22

**Authors:** Xiaopeng Cai, Jiaming Zhou, Jingwen Deng, Zhi Chen

**Affiliations:** 1 State Key Laboratory for Diagnosis and Treatment of Infectious Diseases, National Clinical Research Center for Infectious Diseases, Collaborative Innovation Center for Diagnosis and Treatment of Infectious Diseases, The First Affiliated Hospital, Zhejiang University School of Medicine, 79# Qingchun Road, Hangzhou, 310003 China; 2 Key Laboratory of Disease Proteomics of Zhejiang Province, Department of Pathology, Zhejiang University School of Medicine, Hangzhou, 310058 China; 3 Women’s Hospital, Zhejiang University School of Medicine, Hangzhou, 310058 China

**Keywords:** SMARCC1, HCC, Survival, Hub genes, Immune infiltrations, Therapeutic target

## Abstract

**Background:**

Epigenetic alterations contribute greatly to metastasis and dissemination in hepatocellular carcinoma (HCC). SMARCC1, as a SWI/SNF chromatin remodeling factor, has been reported to play important roles in many cancers. For the first time, with the bioinformatics analysis and wet-bench experiments, we explored the biological significance of SMARCC1 and its potential as putative therapeutic target in HCC.

**Methods:**

The mRNA expression profiles and prognostic value of SMARCC1 were analyzed in the Oncomine, UALCAN and Kaplan–Meier Plotter databases. The expression of SMARCC1 and associated clinicopathological factors were further evaluated using a tissue microarray. Differentially expressed genes associated with SMARCC1 in HCC were obtained and analyzed via the LinkedOmics and GEPIA databases and Cytoscape software. To verify the important role of SMARCC1 in HCC, we knocked down and overexpressed SMARCC1 in different hepatic cell lines and conducted several functional experiments. Then, we evaluated the mutation profiles and transcriptional regulators of SMARCC1 using the cBioPortal, COSMIC, CistromeDB and TCGA databases. Finally, we addressed the relationship of SMARCC1 expression with immune cell infiltration via TIMER database analysis.

**Results:**

Through data mining and tissue microarray verification, we found that the protein and mRNA levels of SMARCC1 are high in tumor tissues, which has remarkable diagnostic value in HCC patients. SMARCC1 and its hub genes showed prognostic value in HCC. Furthermore, we confirmed that SMARCC1 influenced the proliferation, migration, and invasion of HCC cells. Moreover, correlation analyses revealed that SMARCC1 expression was positively correlated with ZBTB40 transcription factors and negatively correlated with the DNA methylation level. Overall, we found that SMARCC1 affects immune infiltration and plays a tumor-promoting role in HCC.

**Conclusions:**

SMARCC1 is overexpressed and is a putative prognostic predictor in HCC. Due to the tumor-promoting role of SMARCC1, treatments inhibiting DNA methyltransferases and transcription factors or weakening the role of SMARCC1 in immune infiltration might improve the survival of HCC patients.

**Supplementary Information:**

The online version contains supplementary material available at 10.1186/s12935-021-02413-w.

## Background

Hepatocellular carcinoma (HCC), which comprises 75%-85% of primary liver cancer cases, is one of the most frequent human malignancies in the world. It is the third leading cause of cancer-related death globally [1]. HCC patients typically have a poor prognosis because of late diagnosis. Many patients with advanced stages of HCC miss the optimal period for effective treatment, and only 1/3 of newly diagnosed patients are eligible for curative therapies. In addition, metastasis and recurrence are the main obstacles limiting improvements in the prognosis and treatment outcomes of HCC [2]. These processes are believed to result from the accumulation of multiple genetic and epigenetic alterations [3]. Epigenetic changes contribute more to HCC metastasis and dissemination than genetic alterations [4]. Chromatin remodeling factors have gained much attention because of their essential roles in dynamically regulating gene expression. Through disruption of nucleosomes, the SWItch/Sucrose Non Fermentable (SWI/SNF) complex is involved in chromatin remodeling [5].

The SWI/SNF complex contains 5 core subunits and 7–15 accessory subunits and functions by interfering with histone-DNA contacts using energy from ATP [6]. It can either activate or suppress endogenous gene expression by binding to transcriptional regulators to exposed DNA [7]. Accumulating evidence shows that the SWI/SNF complex plays an important role in the development and prognosis of various cancers, as almost 25% of all cancers harbor mutations in one or more of these subunits [8]. As a core subunit of the complex, SWI/SNF-related, matrix-associated, actin-dependent regulator of chromatin subfamily C member 1 (SMARCC1) is worthy of detailed study. Although the upregulation of other subunits of SWI/SNF, including SMARCD1, SMARCA4 and ARID1A, has been observed in HCC patients and shown to be associated with poor overall survival (OS) [9–12], the role of SMARCC1 is not yet clear. In prostate cancer and colorectal carcinoma, SMARCC1 was suggested to contribute, at least partially, to tumorigenesis [13, 14]. Thus, we assessed whether SMARCC1 is involved in the development of HCC.

By utilizing a panel of online bioinformatics tools, we found that the expression of SMARCC1 was significantly upregulated in HCC tissue compared with benign liver tissue, which was confirmed by our tissue microarray of SMARCC1 in a local HCC cohort. Moreover, this upregulation was indicated to be related to poor OS. Then, we performed SMARCC1 knockdown and overexpression in HCC cell lines and confirmed the relevant tumor-promoting functions of SMARCC1 in vitro. Finally, we identified a positive correlation between SMARCC1 and tumor-infiltrating immune cells. This is the first study of the expression of SMARCC1 and its prognostic significance and associations with immune infiltrates in HCC. This work suggests that SMARCC1 is also a putative therapeutic target in HCC.

## Methods

### Expression of SMARCC1 in HCC

We searched ‘SMARCC1’ as the gene symbol in the Oncomine database. SMARCC1 expression values (log2 median-centered ratio) from four Gene Expression Omnibus (GEO) sets (including Roessler liver, GSE14520; Wurmbach liver, GSE6764; Roessler liver2, GSE14520; Chen liver, GSE3500) [15–17] were obtained and graphed using GraphPad Prism 8.0 software. Next, subgroup analysis of SMARCC1 expression was conducted using the UALCAN [18]. The UALCAN database includes the sequencing data of 371 liver hepatocellular carcinoma (LIHC) tissues and 50 normal tissues from The Cancer Genome Atlas (TCGA) database, along with analysis tools. In addition, we further verified the protein expression level of SMARCC1 using the Human Protein Atlas (HPA) database [19, 20].

### Survival analysis of SMARCC1 in HCC

SMARCC1 expression and OS in HCC patients were evaluated using Kaplan–Meier Plotter based on the TCGA database [21]. OS, progression-free survival, recurrence-free survival and disease-specific survival were designated at the endpoints of observation. Then, the OS of different clinical subgroups was analyzed.

### Validation of the expression profile and prognostic value

A tissue microarray was purchased from Outdo Biotech (Shanghai, China), which contains 90 liver tumors and 90 adjacent tissues. Detailed clinical information was collected and is listed in Table 1. Then, we performed immunohistochemical (IHC) staining on these 180 tissues, with a primary anti-SMARCC1 antibody (dilution 1:300, GTX114777, GeneTex, Texas, USA) and a secondary antibody of anti-rabbit (dilution 1:1000, #18653, Cell Signaling Technologies, Massachusetts, USA). Then the tissue microarray was digitized using Aperio scanners (Aperio XT, LEICA, Germany). The results were assessed blindly by two independent pathologists according to the staining area and intensity.

**Table 1:** Correlation between SMARCC1 expression and clinicopathological characteristics in HCC patients

	Variables	SMARCC1 expression		Total	χ^2^	P value
		low	high			
Age (year)	< 50	14	25	39	0.163	0.687
	≥ 50	22	33	55		
Sex	Female	3	11	14	1.981	0.159
	Male	33	47	80		
Grade	I–II	29	31	60	8.247	0.004
	III	6	27	33		
T stage	I–II	27	33	60	3.153	0.076
	III–IV	9	25	34		
TNM stage	I–II	27	33	60	3.153	0.076
	III–IV	9	25	34		
Cirrhosis	Negative	7	12	19	0.001	0.981
	Positive	26	44	70		
Hepetitis	Negative	1	4	5	0.154	0.695
	Positive	35	54	89		
AFP^#^	Negative	16	17	33	2.459	0.117
	Positive	15	33	48		
Cytokeratin-19 ^#^	Negative	29	35	64	4.176	0.041
	Positive	7	23	30		

^#^These results are based on IHC of liver specimens

### Differentially expressed gene (DEG) and hub gene analysis of SMARCC1 in HCC

The LinkedOmics database contains multiomics data of 32 cancer types [22]. We selected 371 TCGA-LIHC samples for analysis. Based on the DEGs mined from the LinkedOmics database, Cytoscape software was used to determine hub genes [23]. The GEPIA database was then employed to verify the correlation between hub genes and SMARCC1 expression [24].

### The malignant behaviors of SMARCC1 in HCC

HCC cell lines (HepG2, Huh7, Hep3B and PLC-8024) were purchased from the Chinese Academy of Sciences. The cells were cultured in DMEM with 10% fetal bovine serum (FBS, Gibco, USA) and grown at 37 °C in an atmosphere of 5% CO_2_.

We used Lipofectamine 3000 (Invitrogen, USA)-transfected HepG2 and Huh7 cells to instantaneously obtain knockdown cells. All transfections were performed according to the manufacturer’s instructions. The siRNA sequences were as follows: siRNA-negative (UUCUCCGAACGAGUCACGUTT), siRNA1 (GGGCUGCUUACAAGUAUAATT) and siRNA2 (GCUGAAGUAUGCUGAAUUATT). These sequences were purchased from GenePharm (Shanghai, China). To obtain stable SMARCC1 knockdown HepG2 and Huh7 cell lines, we used a lentivirus-based short hairpin RNA (shRNA) delivery system, and the targeting sequences were GCGGATTTCAACCAAGAATGA (shRNA1) and GGGACTCGTTAATTACCAAGT (shRNA2). All the steps of a previous study were followed [25]. Then, PLC-8024 cell lines stably expressing SMARCC1 were obtained by transfection with the pCDH-3 × flag vector containing SMARCC1 DNA and selected in 10 µg/ml G418 for 14 days. Finally, the mRNA expression level of SMARCC1 was evaluated using qPCR.

Cell Counting Kit-8 (CCK-8, DOJINDO, Japan) was used to evaluate HCC cell ability. HCC cells were seeded evenly into 96‐well plates at 2000 cells/well. Then, we detected cell viability at different time points (0 h, 24 h, 48 h, 72 h and 96 h). We used Transwell migration and Transwell invasion assays to detect the migration and invasion ability of HCC cells. HCC cell suspension was plated into the upper chamber (Costar, USA, 8.0 µm with Size 24 Cluster Plate) with serum-free medium, and the medium of the lower chamber contained 10% FBS. Fifty microliters of 1:50 diluted extracellular matrix gel (Gibco, USA) was added to the upper chamber for the invasion assays but not migration assays. A total of 2 × 10^5^ HepG2 cells, 1 × 10^5^ Huh 7 cells, 1 × 10^5^ PLC-8024 cells and 1 × 10^5^ Hep3B cells were seeded for migration and invasion assays. After incubation for 24 h (Huh7 and PLC-8024), 72 h (HepG2) and 96 h (Hep3B), the cells in the upper chamber were fixed with 4% paraformaldehyde and stained with crystal violet. We observed 6 fields per chamber to count invaded cells at a magnification of 100 ×. All the assays were performed in triplicate.

### SMARCC1 mutation and transcriptional regulation analysis in HCC

The mutant frequency of SMARCC1 in HCC was evaluated using the cBioPortal database [26, 27]. Mutations in SMARCC1 in HCC were further validated in the COSMIC database [28, 29]. To shed light on the mechanism of SMARCC1 regulation in HCC, we conducted transcription factor prediction using CistromeDB [30, 31] and DNA methylation analysis based on the TCGA database.

### Immune infiltration analysis of SMARCC1 in HCC

Next, the associations between SMARCC1 and immune infiltrates were analyzed using the TIMER database [32]. Moreover, we investigated the influence of SMARCC1 expression on immune cells using the single-sample gene set enrichment analysis (ssGSEA) immune infiltration algorithm based on RNA-seq data mined from the TCGA [33, 34].

### Statistical analysis

Data are summarized as the mean ± SEM. Differences between 2 groups were evaluated using Student’s t test, and a P value < 0.05 was used as the threshold to identify significant differences. Partial results were analyzed using GraphPad Prism 8.0 software.

## Results

### Elevated expression of SMARCC1 in HCC

**Fig. 1:**
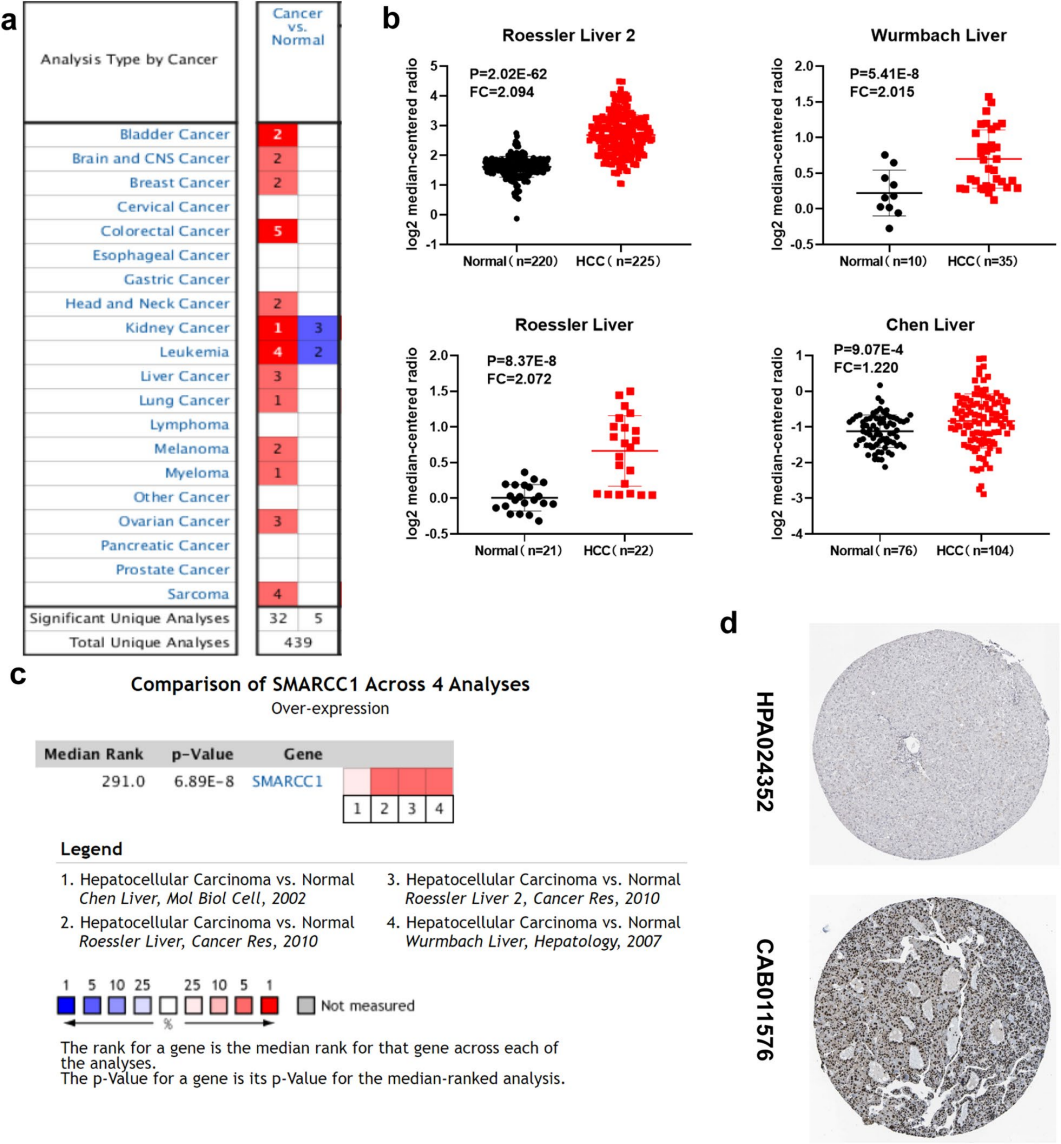
The expression profile of SMARCC1 in HCC. **a** SMARCC1 is overexpressed in several types of cancers (increased expression-red colour, decreased expression-blue colour). **b** SMARCC1 is overexpressed in four datasets of HCC. **c** Comparison of SMARCC1 across the four studies. **d** Protein expression of SMARCC1 is elevated in HCC tissues from HPA database

**Fig. 2:**
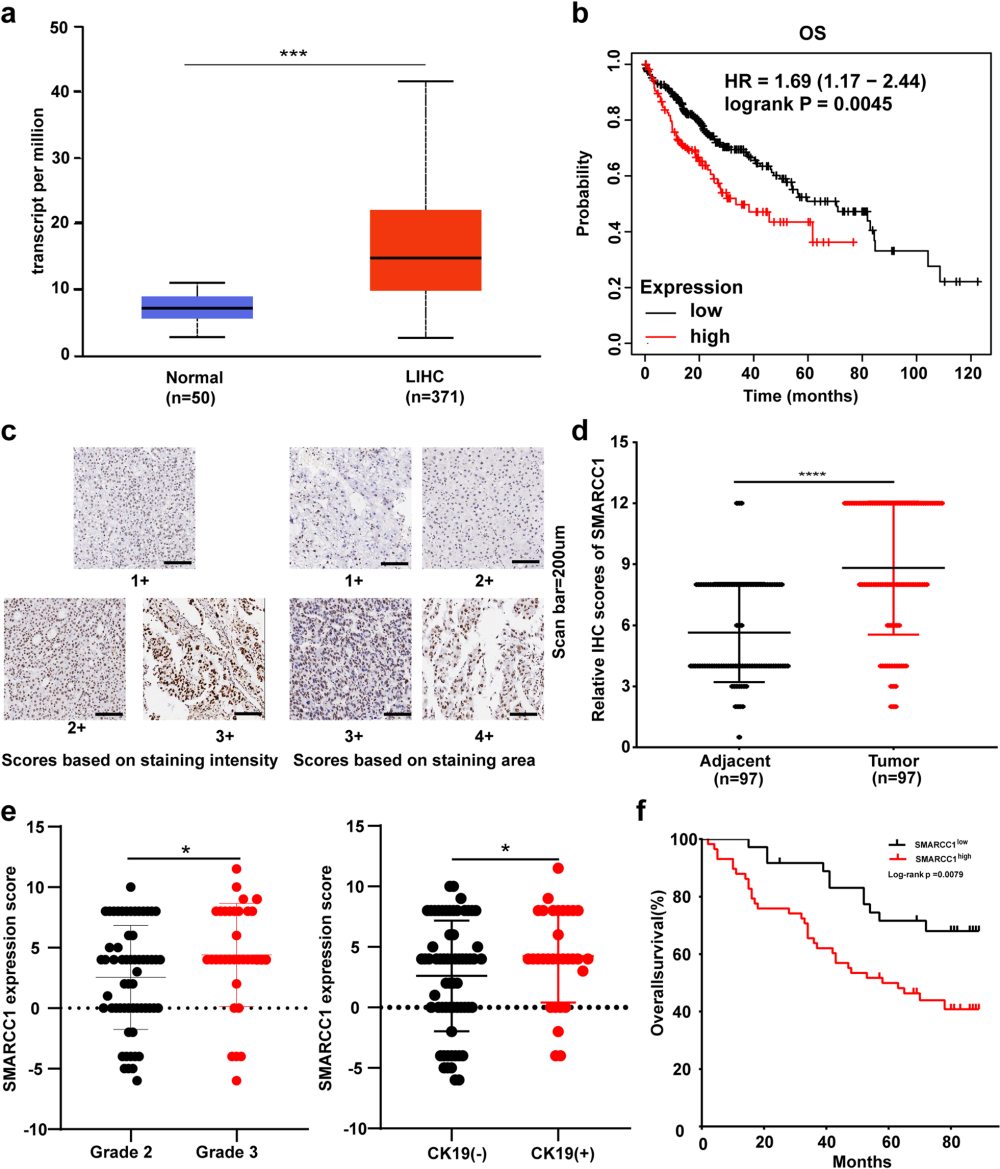
The expression profile and prognostic values of SMARCC1 in HCC. **a** mRNA expression of SMARCC1 in normal tissues and LIHC samples from TCGA database. **b**The prognostic values of SMARCC1 in HCC from Kaplan–Meier Plotter online tool. **c** Representative of immunohistochemical staining intensity and positive rate of SMARCC1 in HCC tissues from a tissue microarray (scan bar = 200um). **d** Expression of SMARCC1 protein in tumor tissues compared to adjacent tissues from a tissue microarray (scan bar = 200um). **e** Relationships between SMARCC1 expression and tumor grades or cytokeratin-19 (CK19) status in a local cohort of HCC patients. **f** Overall survival analysis in the cohort based on SMARCC1 expression levels in a local cohort of HCC patients. The values are expressed as mean ± SEM, ****P < 0.0001, ***P < 0.001, **P < 0.01 and *P < 0.05

Based on the Oncomine database, we screened the mRNA expression profiles of SMARCC1 across a number of studies for different types of tumors (normal vs. cancer), including colorectal cancer, leukemia and sarcoma (Fig. 1a). Next, we analyzed the expression of SMARCC1 in HCC samples from 4 datasets (Roessler 2, Wurmbach, Chen and Roessler liver) [15–17]. The results showed that the mRNA expression of SMARCC1 in liver tumor tissues was significantly upregulated in all four studies (Fig. 1b). In addition, a comparison of SMARCC1 across these 4 studies indicated the existence of interpatient variations in SMARCC1 expression at the mRNA level (Fig. 1c). We further validated the upregulation of SMARCC1 protein expression in liver tumor tissues using the HPA database. Stronger SMARCC1-positive staining was found in liver tumor tissues (HCC patient ID 2279) than in a normal control liver tissue (ID 3402), which had no sign of positive signal (Fig. 1d). To elucidate the expression patterns of SMARCC1 in HCC patients, we employed the UALCAN database. Overall, the expression of SMARCC1 was significantly upregulated in liver tumor samples (Fig. 2a). We observed intriguing variations, which were quite significant among patients grouped by age, race, HCC stage, and tumor grade (Additional file 1: Fig. S1). For example, SMARCC1 mRNA expression was much higher in patients in the grade III–IV, stage I-III and regional lymph node metastasis groups than in other groups. Overall, we concluded that elevated expression of SMARCC1 is correlated with HCC progression.

### Survival results and multivariate analysis in HCC patients

To investigate the prognostic significance of SMARCC1 in HCC, we analyzed survival curves using the Kaplan–Meier Plotter database. We found that a high expression level of SMARCC1 indicated poor OS (Fig. 2b). The prognostic value of SMARCC1 was achieved not only in patients with high SMARCC1 expression but also in different subgroups (female patients, white patients, alcohol consumers and patients without hepatitis virus infection) (Additional file 2: Fig. S2). In conclusion, high SMARCC1 expression can serve as a prognostic biomarker in HCC.

### Validation of the expression profile and prognostic value of SMARCC1

According to the above bioinformatics and data analysis results, we further assessed the correlation between the clinical characteristics of HCC patients and SMARCC1 expression experimentally using a tissue microarray. According to the area and intensity of staining, we defined several different staining patterns (Fig. 2c). Consistent with the findings above, SMARCC1 protein expression was elevated in HCC tissues compared with paired normal liver tissues (Fig. 2d). Next, we investigated the correlation between SMARCC1 expression and clinicopathological characteristics (Table 1). We found that patients with higher grade tumors showed increased SMARCC1 expression scores and that a similar pattern was observed for patients with high cytokeratin-19 (CK19) expression (Fig. 2e). CK19 is a biomarker of HCC stem cells [35], and its expression can be used to predict the early postoperative recurrence of HCC due to increased invasiveness [36]. These facts compelled us to address the relationship between SMARCC1 and survival in our HCC cohort. According to OS curve analysis, patients with elevated SMARCC1 expression had a poorer prognosis (Fig. 2f). Univariable analysis demonstrated that high SMARCC1 expression, T stage and tumor-node-metastasis (TNM) stage were potential risk factors for decreased OS in HCC (Table 2). According to multivariable analysis, high SMARCC1 expression and TNM stage were independent predictors of OS in our cohort study. Overall, we concluded that SMARCC1 is a putative novel predictor for prognosis in HCC.

**Table 2:** Univariate and multivariate analyses of the factors correlated with Overall survival of Liver carcinoma patients

Variables	Univariate analysis			Multivariate analysis		
	HR	95%CI	P value	HR	95%CI	P value
SMARCC1 Expression	2.458	1.239–4.873	0.010	2.554	1.271–5.133	0.008
Age	1.150	0.626–2.110	0.652			
Sex	1.013	0.451–2.272	0.976			
Grade	0.880	0.467–1.660	0.694			
T stage	2.362	1.302–4.286	0.005	0.422	0.125–1.430	0.166
TNM stage	1.958	1.391–2.756	0.000	3.151	1.501–6.613	0.002
cirrhosis	1.788	0.931–3.434	0.081			
Hep	2.434	0.335–17.693	0.379			
AFP^#^	1.236	0.641–2.384	0.528			
CK19^#^	1.252	0.670–2.338	0.481			

^#^These results are based on IHC of liver specimens

### Genes correlated with SMARCC1 in HCC

**Fig. 3:**
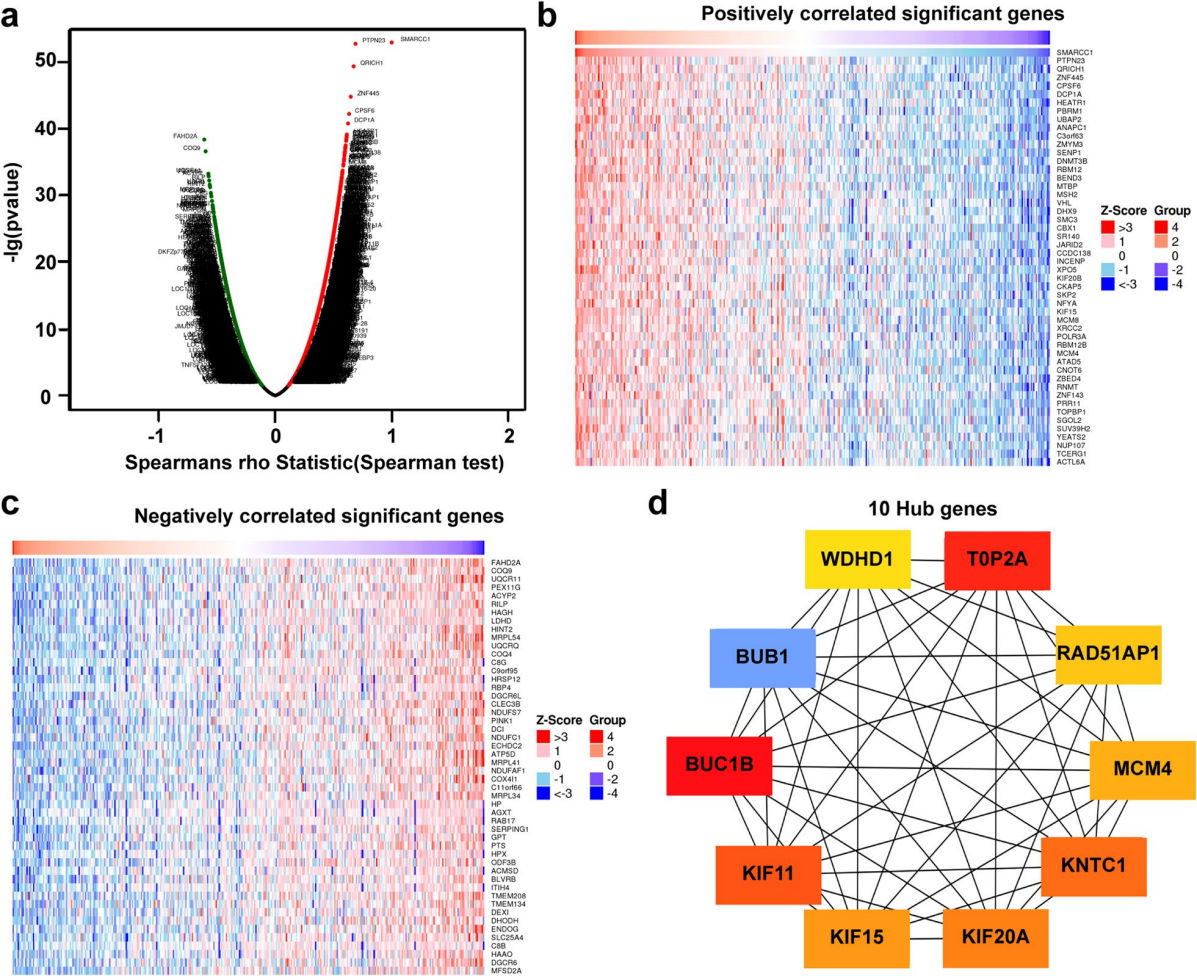
Genes associated with SMARCC1 expression in HCC. **a** Correlations between SAMRCC1 and differently expressed genes from LinkedOmics database. **b**, **c** Heat maps show the genes that are positively or negatively correlated with SMARCC1 (Top 50 genes). **d** The 10 hub genes of SMARCC1

We evaluated the DEGs correlated with SMARCC1 in HCC using the LinkedOmics database. As shown in the volcano map (Fig. 3a), the positively related genes converged on the right of 0 (positive values), and the negatively related genes converged on the left (negative values). Based on the Spearman test, the top 50 positively and negatively related genes were identified and are shown in the heatmaps (Fig. 3b, c). Then, we selected the top 50 genes positively related to SMARCC1 for further analysis. The 50 genes were input into Cytoscape software, and the top 10 hub genes of the network (ranked by degree) were obtained using cytoHubba (Fig. 3d). The 10 hub genes were BUB1, BUB1B, KIF11, KIF15, KIF20A, KNTC1, MCM4, RAD51AP1, TOP20A and WDHD1. The expression of the 10 hub genes was confirmed to be significantly correlated with SMARCC1 using the GEPIA database (Additional file 3: Fig. S3). We evaluated the prognostic significance of the top 10 hub genes using Kaplan–Meier Plotter. All 10 genes were significantly associated with poor OS, especially KIF20A (HR = 2.33) (Additional file 4: Fig. S4). Based on the results, we concluded that the associations between SMARCC1 and its hub genes are a contributing factor for its prognostic value in HCC.

### The malignant behaviors of SMARCC1 in HCC

**Fig. 4:**
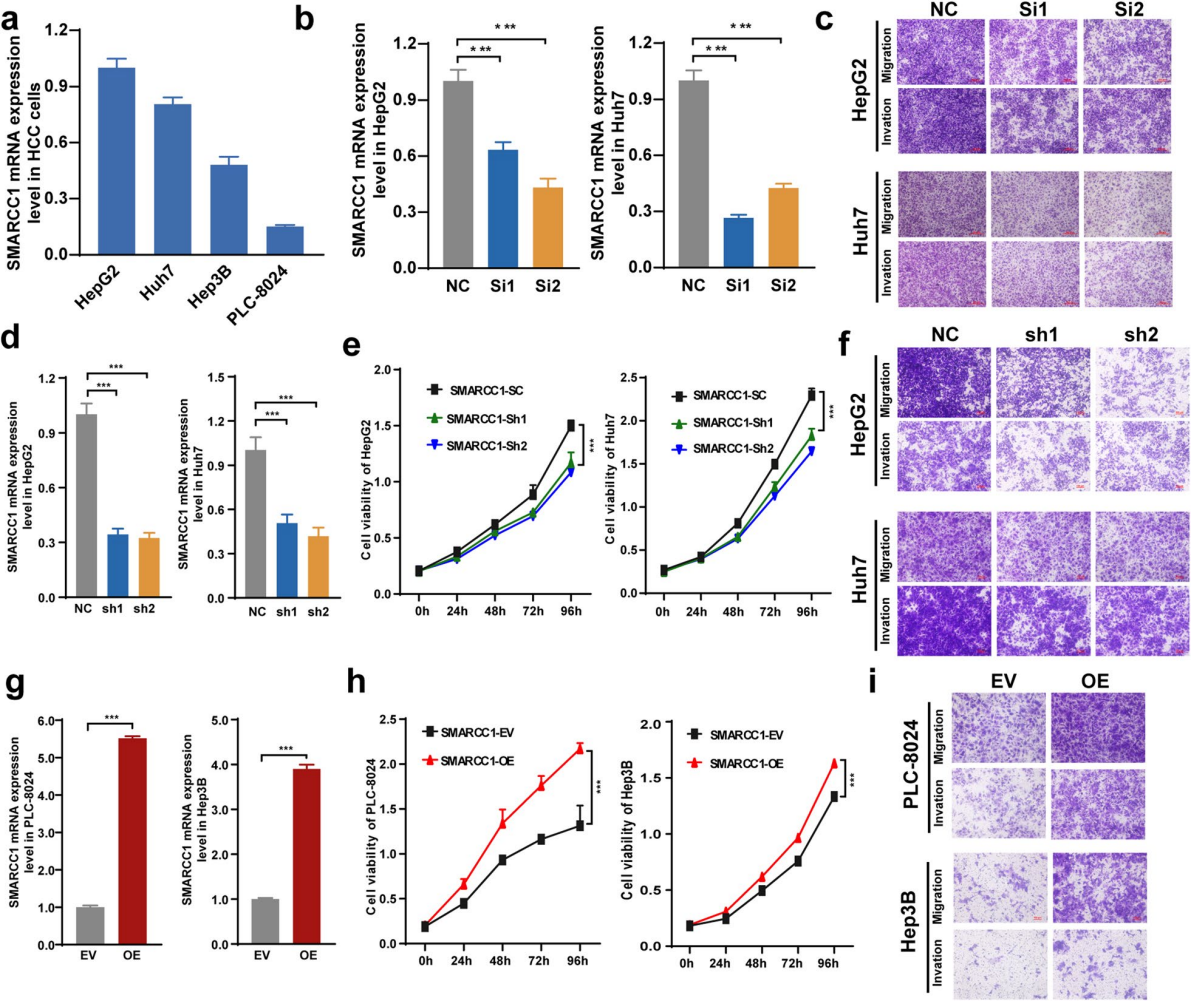
The malignant behaviors of SMARCC1 in HCC. **a** mRNA expression of SMARCC1 in 4 HCC cell lines. **b** mRNA expression of SMARCC1 in HepG2 and Huh7 cells after si-RNA transfection. **c** The migration and invasion ability of HepG2 and Huh7 cells after si-RNA transfection. **d** mRNA expression of SMARCC1 in HepG2 and Huh7 cells after sh-RNA transfection. **e** The cell viability of HepG2 and Huh7 after sh-RNA transfection. **f** The migration and invasion ability of HepG2 and Huh7 cells after sh-RNA transfection. **g** mRNA expression of SMARCC1 of PLC-8024 and Hep3B after sh-RNA transfection. **h** The cell viability of PLC-8024 and Hep3B after over-expressed. **i** The migration and invasion ability of PLC-8024 and Hep3B after over-expressed. The values are expressed as mean ± SEM, ****P < 0.0001, ***P < 0.001, **P < 0.01 and *P < 0.05

To verify that SMARCC1 is involved in tumorigenesis and the development of HCC, we conducted several cellular function analyses. We studied the mRNA expression of SMARCC1 in 4 HCC cell lines (Fig. 4a). Then, SMARCC1 was knocked down instantly and stably in HepG2 and Huh7 cells with high SMARCC1 expression and overexpressed in Hep3B and PLC-8024 cells with low SMARCC1 expression. HepG2 and Huh7 cells with SMARCC1 knockdown showed a decrease in proliferation, migration and invasion ability (Fig. 4b–f). In contrast, the proliferation, migration and invasion activities of Hep3B and PLC-8024-overexpressing cells were elevated (Fig. 4g–i). Collectively, these results demonstrated that SMARCC1 is involved in the malignancy of HCC.

### Mutant and transcriptional regulation analysis of SMARCC1 in HCC

**Fig. 5:**
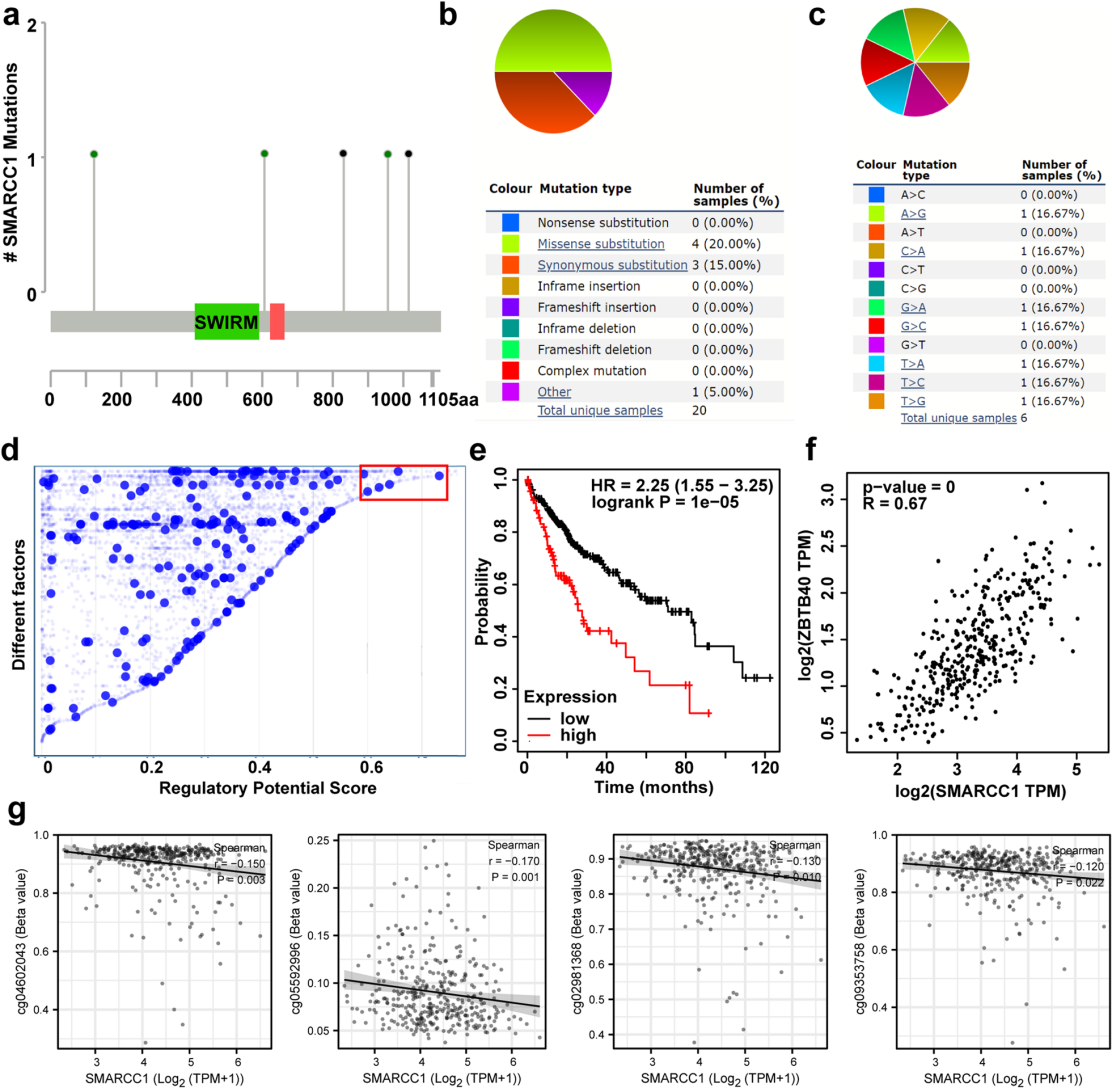
Mutant and transcriptional regulation analysis of SMARCC1 in HCC. **a** The schematic representation of SMARCC1 mutations in HCC. **b**, **c** The mutation types of SMARCC1 (%) in HCC. **d** Transcription factors with high regulatory potential in HepG2 cell lines from Cistrome DB database (10 k distance to TSS). **e** The prognostic values of ZBTB40 in HCC from Kaplan‐Meier Plotter online tool. **f** The relevance of SMARCC1 expression in relation to ZBTB40 expression in HCC from GEPIA database. **g** Relationships of SMARCC1 with DNA methylation in HCC based on TCGA database

To determine the reason SMARCC1 expression is elevated in HCC, we conducted gene mutation and transcription regulation analyses. We found 5 mutations of SMARCC1, including 3 missense mutations and 2 truncating mutations, in HCC patients (Fig. 5a). The somatic mutant frequency of SMARCC1 in HCC was only 0.5%. Then, we verified that the mutation rate of SMARCC1 was 2.1% in HCC patients using the COSMIC database. The most common type of mutation was missense substitution, and the A > G, C > A, G > A, G > A, G > C, T > A, T > C and T > G substitution mutations occurred in equal frequencies (Fig. 5b, c).

To determine the mechanism involved in the regulation of SMARCC1 in HCC. We first performed transcription factor prediction using the Cistrome DB database. We assessed the HCC line HepG2 and found that CEBPB, POLR2A, ZBTB40, NR2F6 and RCOR1 possessed regulatory potential in HepG2 cells (Fig. 5d). Only ZBTB40 was positively correlated with poor prognosis and SMARCC1 expression in HCC (Fig. 5e, f), and the other 4 transcription factors had inconsistent correlations (Additional file 5: Fig. S5). However, the regulatory role of ZBTB40 in SMARCC1 needs further experimental verification. Methylation of the DNA promoter is an epigenetic mechanism that regulates gene expression. The association between SMARCC1 expression and methylation of SMARCC1 in HCC patients was detected based on the TCGA-LIHC dataset. As shown in Fig. 5g, SMARCC1 was negatively correlated with cg04602043, cg05592996, cg02981368 and cg09353758. However, we did not detect correlations between SMARCC1 and the 10 other SMARCC1 CpG sites (Additional file 6: Fig. S6). Collectively, these results suggest that ZBTB40 and DNA methylation play a considerable role in HCC processes by regulating SMARCC1 expression.

### The relationship of immune infiltrates and SMARCC1 in HCC

**Fig. 6:**
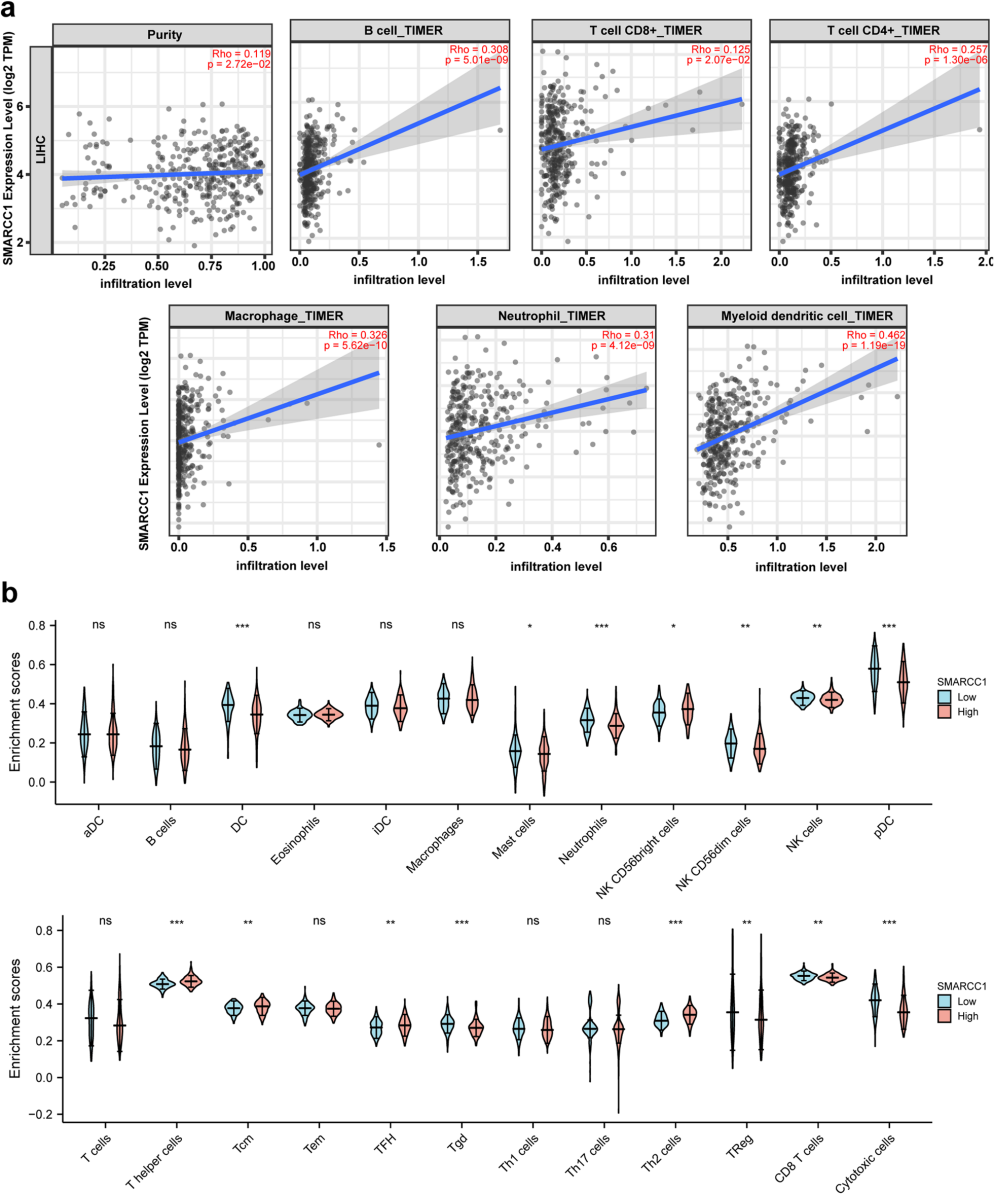
SMARCC1 was associated with immune infiltration in HCC. **a** The correlation between SMARCC1 and tumor purity, B cell, T cells CD8 + , T cells CD4 + , macrophage, neutrophil and myeloid dendritic cell. **b** The infiltration profile of SMARCC1^high^ and SMARCC1^low^ in HCC

Finally, we investigated the relationship of immune infiltrates and SMARCC1 using the TIMER database. Our work showed that SMARCC1 was significantly positively associated with the infiltration of 6 immune cell types, especially B cells, CD4 + T cells and myeloid dendritic cells (Fig. 6a). Furthermore, we investigated the potential correlations between SMARCC1 and a panel of marker genes representative of the 6 immune cell types. SMARCC1 was clearly positively correlated with all listed gene markers (Table 3). The top 5 relevant gene markers were QRSL1, NRP1, STAT1, ITGAX and STAT5A. Moreover, we tried to determine whether SMARCC1 influenced the immune microenvironment. The 374 HCC samples from the TCGA-LIHC dataset were split into 2 groups, namely, the high expression group (187 samples) and the low expression group (187 samples) (Fig. 6b). The infiltration levels of CD8 + T cells, NK cells and dendritic cells were decreased in the high SMARCC1 expression group. These results collectively demonstrated that SMARCC1 is involved in immune infiltration during the progression and development of HCC.

**Table 3:** Correlation between SMARCC1 and immune cells’ gene markers in HCC

Cells subtypes	Markers	Correlation	*P*-value	adj. *P*-value
B cells	CD19	0.266	0.000	0.000
	CD79A	0.227	0.000	0.000
T cells (general)	CD2	0.243	0.000	0.000
	CD3D	0.226	0.000	0.000
	CD3E	0.270	0.000	0.000
CD8 + T cells	CD8A	0.244	0.000	0.000
	CD8B	0.176	0.001	0.010
CD4 + T cells	CD4	0.234	0.000	0.000
	QRSL1	0.569	0.000	0.000
	STAT1	0.510	0.000	0.000
	STAT4	0.248	0.000	0.000
	STAT5A	0.442	0.000	0.000
	STAT6	0.343	0.000	0.000
	TBX21	0.204	0.000	0.000
Tumor associated macrophages	CCL2	0.230	0.000	0.000
	CD68	0.320	0.000	0.000
	IL10	0.380	0.000	0.000
Type I macrophages	IRF5	0.421	0.000	0.000
	NOS2	0.066	0.224	0.319
	PTGS2	0.330	0.000	0.000
Type II macrophages	CD163	0.276	0.000	0.000
	MS4A4A	0.254	0.000	0.000
	VSIG4	0.240	0.000	0.000
Neutrophil	CCR7	0.224	0.000	0.000
	ITGAM	0.398	0.000	0.000
Dendritic cells	CD1C	0.286	0.000	0.000
	HLA-DPB1	0.247	0.000	0.000
	HLA-DQB1	0.179	0.001	0.004
	HLA-DRA	0.303	0.000	0.000
	ITGAX	0.450	0.000	0.000
	NRP1	0.531	0.000	0.000

Currently, immune checkpoint inhibitors (ICIs), such as those targeting CTLA-4, PD-1, and the PD-L1 axis, have shown good prospects in various types of malignant tumors [37]. Based on the fact that SMARCC1 is associated with immune infiltration, we explored the association of SMARCC1 expression with the abundance of several immune checkpoints. The results showed that SMARCC1 expression was obviously associated with the expression of immune checkpoints on immune cells and cancer cells, including PD-1, PD-L1, PD-L2, and CTLA-4 (Table 4).

**Table 4:** Correlation between SMARCC1 and immune checkpoints expression

immune cells	None		Purity		tumor cells	None		Purity	
	Rho	P	Rho	P		Rho	P	Rho	P
PD1	0.23	< .001	0.23	< .001	PDL1	0.28	< .001	0.29	< .001
CTLA4	0.20	< .001	0.22	< .001	PDL2	0.10	0.051	0.11	0.031
CD28	0.30	< .001	0.31	< .001	CD80	0.20	< .001	0.20	< .001
LAG3	0.12	0.014	0.14	0.007	CD86	0.28	< .001	0.29	< .001
CD226	0.24	< .001	0.25	< .001	FGL1	− 0.23	< .001	− 0.21	< .001
TIGIT	0.21	< .001	0.24	< .001	LGALS3	0.19	< .001	0.20	< .001
CD96	0.18	< .001	0.20	< .001	CD112	0.24	< .001	0.24	< .001
BTLA	0.18	< .001	0.19	< .001	CD115	0.20	< .001	0.22	< .001
VISTA	0.27	< .001	0.27	< .001	HVEM	0.16	0.001	0.16	0.002
TIM3	0.27	< .001	0.29	< .001	CEACAM1	0.11	0.025	0.13	0.009

## Discussion

In HCC, epigenetic alterations contribute greatly to metastasis and dissemination [3]. These alterations directly induce certain changes to chromatin configuration and rearrangement. In other words, chromatin can be remodeled in tumor cells. Among epigenetic-related genes, chromatin remodeling factors have attracted our attention because they can dynamically regulate gene expression.

As a key chromatin remodeling complex, SWI/SNF participates in many cellular signaling pathways, including cell adhesion, cell cycle, apoptosis, DNA repair, cell morphology, and stress responses [5]. Genes encoding this complex have been shown to be very frequently mutated in cancers, accounting for 25% of all cancer-related mutations. Accumulating evidence demonstrates that this complex is likely to play a tumor suppressive role [38]. However, recently, a few specific subunits were indicated to function as oncogenes or prognostic predictors. For example, high expression of SMARCD1, SMARCA4 and ARID1A can promote tumor cell proliferation and invasion, accompanied by poor survival [9–12]. Based on a bioinformatics analysis, BRD9 and ACTL6A were suggested to play oncogenic roles [39].

In HCC patients, several SWI/SNF subunits, such as SMARCD1, SMARCA4 and ARID1A, were upregulated and shown to be associated with poor overall survival [9–12]. Although SMARCC1 is a core subunit, the role of SMARCC1 has not yet been addressed. In our study, SMARCC1 expression was found to be elevated in HCC at both the mRNA and protein levels by systemic data mining and clinical tissue microarray analysis, respectively. High expression of SMARCC1 was related to unfavorable clinical features and poor OS in HCC patients.

We utilized a network analyst algorithm to further explore the associations between SMARCC1 and coexpressed genes. We found that the top 10 hub genes were also remarkably correlated with a poor prognosis in HCC. This may suggest that SMARCC1 is involved in HCC by playing some essential regulatory roles. Notably, some studies demonstrated that SMARCC1 was correlated with proliferation and metastasis in prostate cancer and colorectal carcinoma [13, 14]. We also conducted several functional trials in vitro and demonstrated that SMARCC1 is involved in the malignant behaviors of HCC, including proliferation, migration and invasion activity.

We wondered why SMARCC1 is elevated in HCC patients. We found that SMARCC1 mutation was not the key reason because the mutation rate was low. By evaluating the potential transcription factors and the DNA methylation levels of SMARCC1 in HCC, we found that the transcription factor ZBTB40 and DNA methylation modifications might play a considerable role in HCC processes by regulating SMARCC1 expression. ZBTB40 is an uncharacterized transcriptional regulator that mainly regulates cell commitment, differentiation, and stem cell self-renewal [40]. Recently, a study found that ZBTB40 modulated the phenotype of osteoblast mineralization in vitro [41]. We speculate that ZBTB40 may regulate the expression of SMARCC1 based on bioinformatics mining, which further needs to be verified by experiments. If this regulatory relationship does exist, ZBTB40 inhibitors might also help to improve the treatment outcomes of HCC patients. DNA methylation may play another pivotal role in upregulating SMARCC1 expression, as it was negatively correlated with SMARCC1 expression. We found that SMARCC1 expression was related to its hypomethylation. Altering DNA methyltransferase activity to reduce SMARCC1 expression may be a potential strategy to improve the prognosis of HCC patients.

The tumor immune microenvironment has a distinct influence on the carcinogenesis of HCC [42]. Moreover, immunotherapy, especially ICIs, has been demonstrated to be a crucial method to treat malignant tumors [37]. Therefore, we investigated whether the expression of SMARCC1 correlates with immune infiltration in HCC. Here, we found that SMARCC1 expression had a positive association with immune infiltrates and immune checkpoints. In addition, we determined the difference in immune infiltrates in HCC patients with different expression levels of SMARCC1. Our findings showed that the levels of CD8 + T cells, NK cells and dendritic cells were decreased in the high SMARCC1 expression group. These cells play an important role in tumor clearance. We could conclude that SMARCC1 alters the ability of these cells to clear tumors. We also believe that SMARCC1 can be used as an inhibitor for immunotherapy due to its positive correlation with ICIs.

## Conclusion

Our explorative study demonstrated that elevated expression of SMARCC1 is closely associated with a poor prognosis in HCC patients. SMARCC1 participates in malignant behaviors and influences the immune environment in HCC. Regulating the expression level of SMARCC1 or implementing immunotherapy targeting SMARCC1 might improve the prognosis of HCC patients.

## Supplementary Information

**Additional file 1: Figure 1. **SMARCC1 mRNA expression levels of HCC patients in subgroups with different ages, genders, races, weights tumour stages, tumour grades,metastasis status and TP‐53 mutant.

**Additional file 2: Figure 2. **The survival curve were analyzed in regards to the mRNA expression level of SMARCC1 in subgroups of HCC patients. OS analysis of Male, Asian race, Alcohol consumption, Hepatitis virus infected, Female, White race, Non-alcohol consumption and Non-hepatitis virus infected. OS, overall survival.

**Additional file 3: Figure 3. **The relevance of SMARCC1 gene expression in relation to the 10 hub genes.

**Additional file 4: Figure 4. **The prognostic values of the top 10 hub genes in HCC.

**Additional file 5: Figure 5. **a The prognostic values of the other 4 transcription factors in HCC from Kaplan‐Meier Plotter online tool. b The relevance of SMARCC1 expression in relation to the other 4 transcription factors expression in HCC from GEPIA database.

**Additional file 6: Figure 6. **Relationships of SMARCC1 with the other 10 DNA methylation sites in HCC based on TCGA database.

## Data Availability

The original contributions presented in the study are included in the article/additional material, further inquiries can be directed to the corresponding author/s.

## Abbreviations

SMARCC1: SWI/SNF related, matrix associated, actin dependent regulator of chromatin subfamily C member 1
HCC: Hepatocellular cancer
TMA: Tissue microarray
SWI/SNF: SWItch/sucrose non-fermentable
DEG: Differentially expressed gene
GEPIA: Gene expression profiling interactive analysis
COSMIC: Catalogue of somatic mutations in cancer
TIMER: Tumor immune estimation resource
KEGG: Kyoto encyclopedia of genes and genomes
GO: Gene ontology
PPI: Protein–protein interaction
