# Supplementary figures and images for: Prognostic biomarker SMARCC1 and its association with immune infiltrates in hepatocellular carcinoma
Source: Cancer Cell Int. 2021 Dec 22;21:701. doi: 10.1186/s12935-021-02413-w (PMC8697473; doi:10.1186/s12935-021-02413-w)

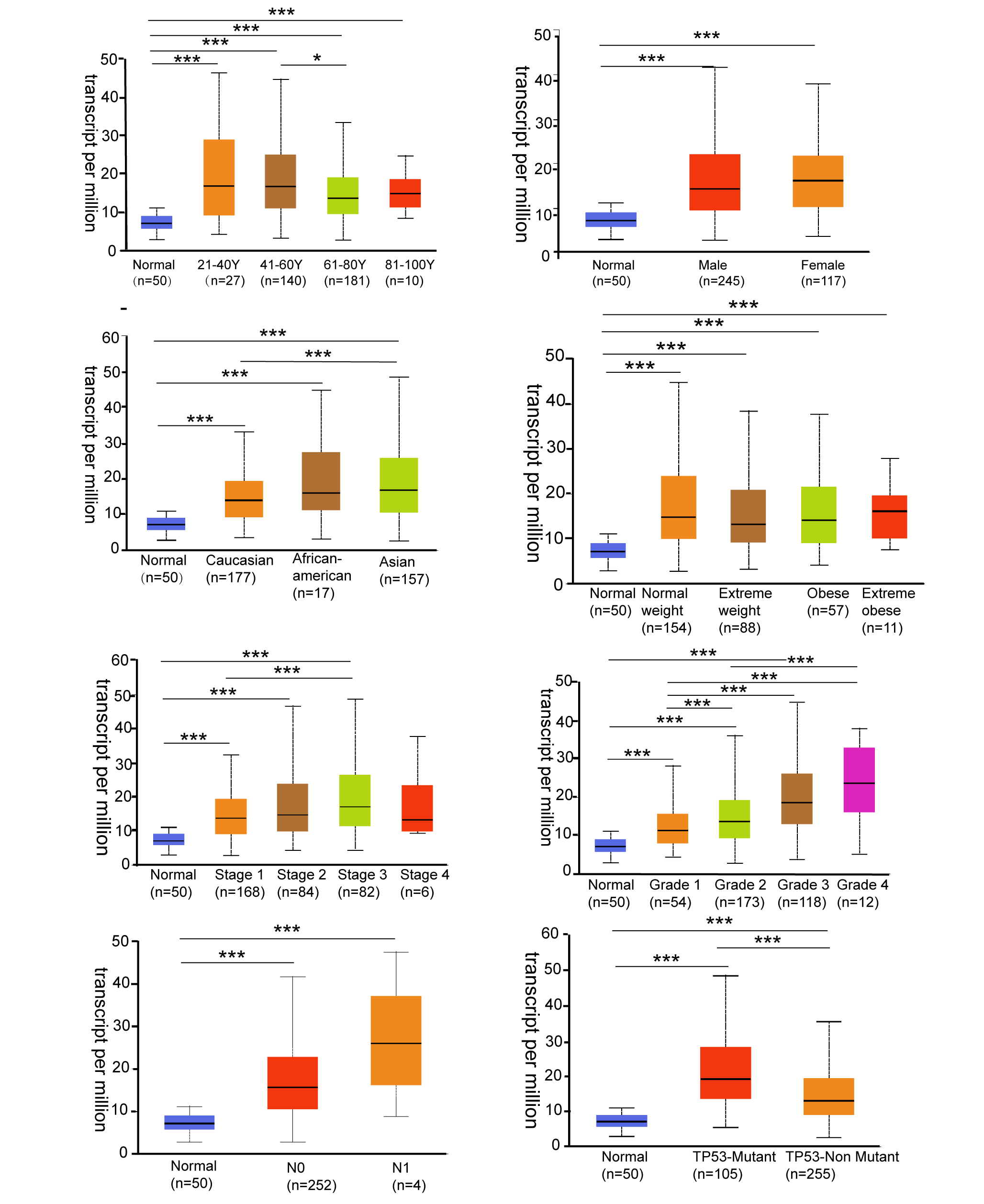

Supplement: Supplementary file 1 — Additional file 1: Figure 1. SMARCC1 mRNA expression levels of HCC patients in subgroups with different ages, genders, races, weights tumour stages, tumour grades,metastasis status and TP‐53 mutant. [file 12935_2021_2413_MOESM1_ESM.tif]

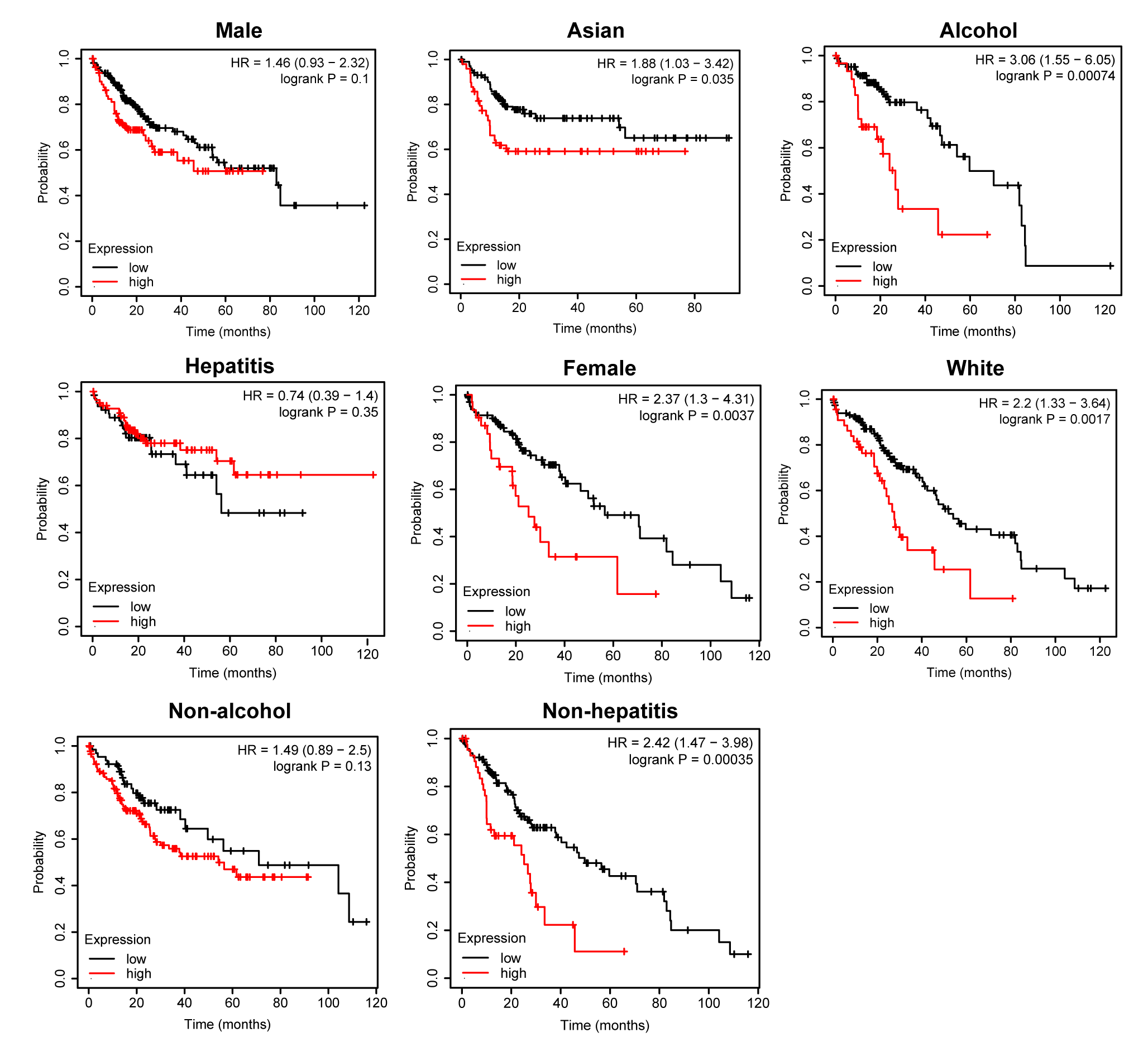

Supplement: Supplementary file 2 — Additional file 2: Figure 2. The survival curve were analyzed in regards to the mRNA expression level of SMARCC1 in subgroups of HCC patients. OS analysis of Male, Asian race, Alcohol consumption, Hepatitis virus infected, Female, White race, Non-alcohol consumption and Non-hepatitis virus infected. OS, overall survival. [file 12935_2021_2413_MOESM2_ESM.tif]

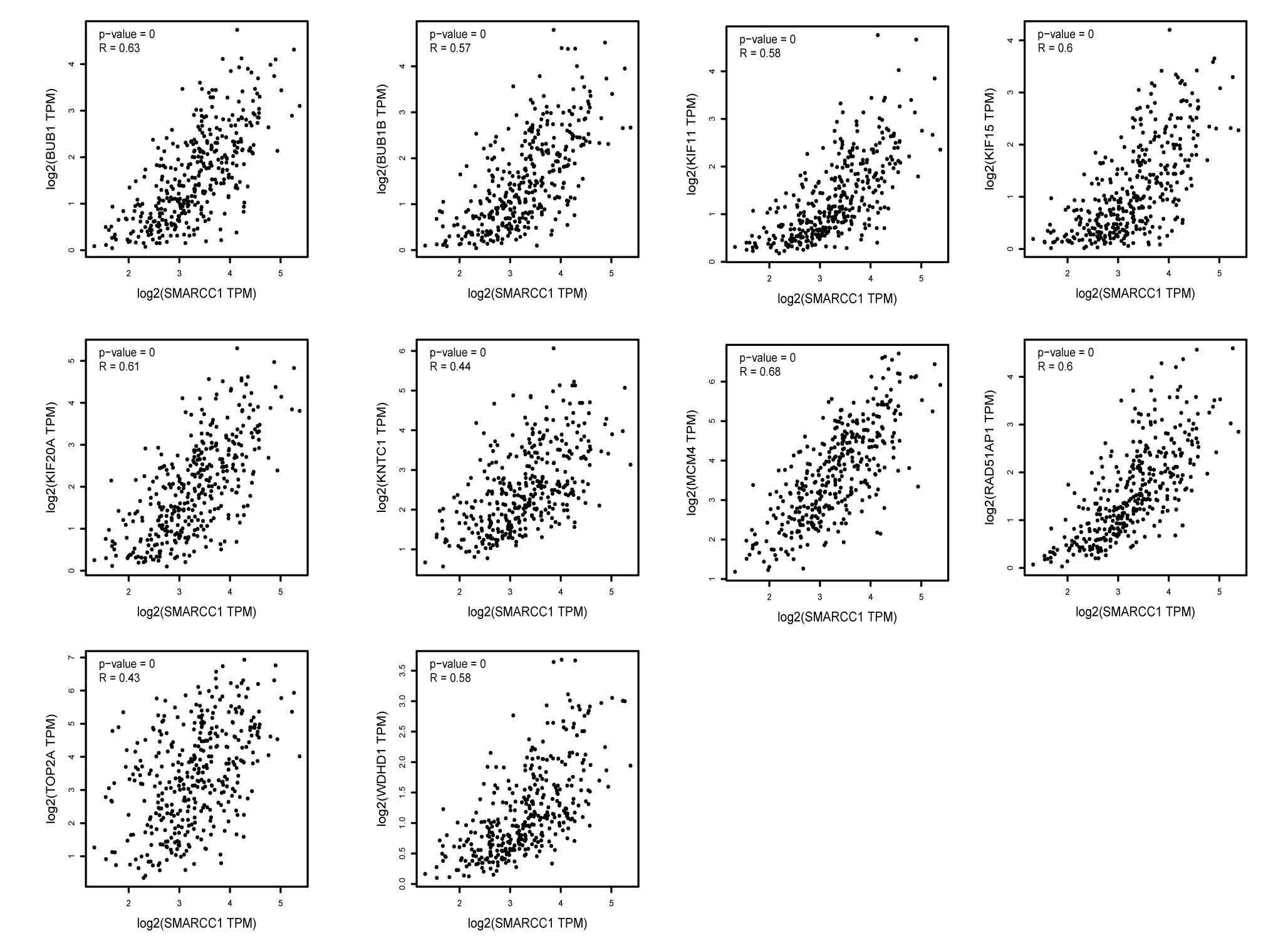

Supplement: Supplementary file 3 — Additional file 3: Figure 3. The relevance of SMARCC1 gene expression in relation to the 10 hub genes. [file 12935_2021_2413_MOESM3_ESM.tif]

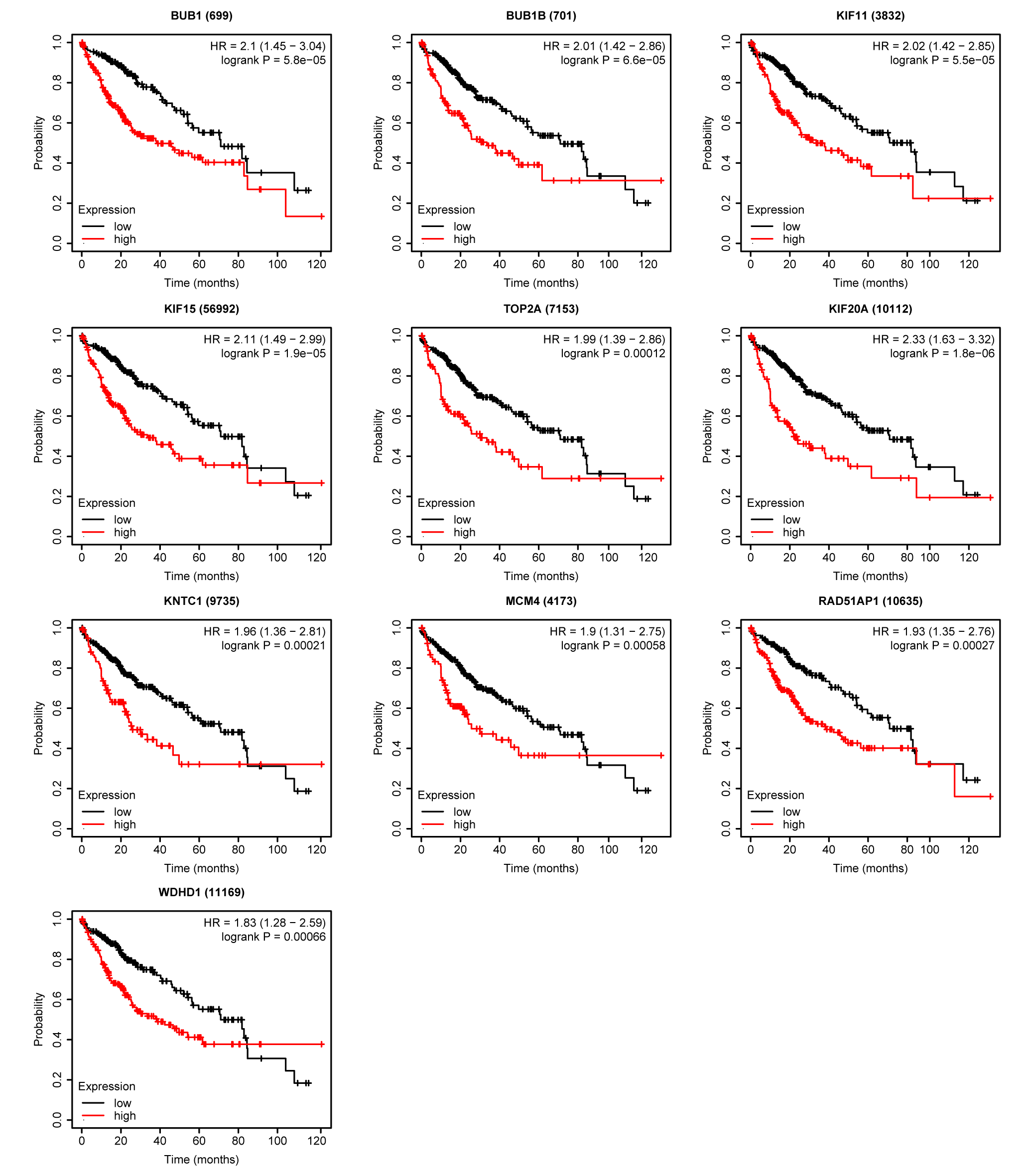

Supplement: Supplementary file 4 — Additional file 4: Figure 4. The prognostic values of the top 10 hub genes in HCC. [file 12935_2021_2413_MOESM4_ESM.tif]

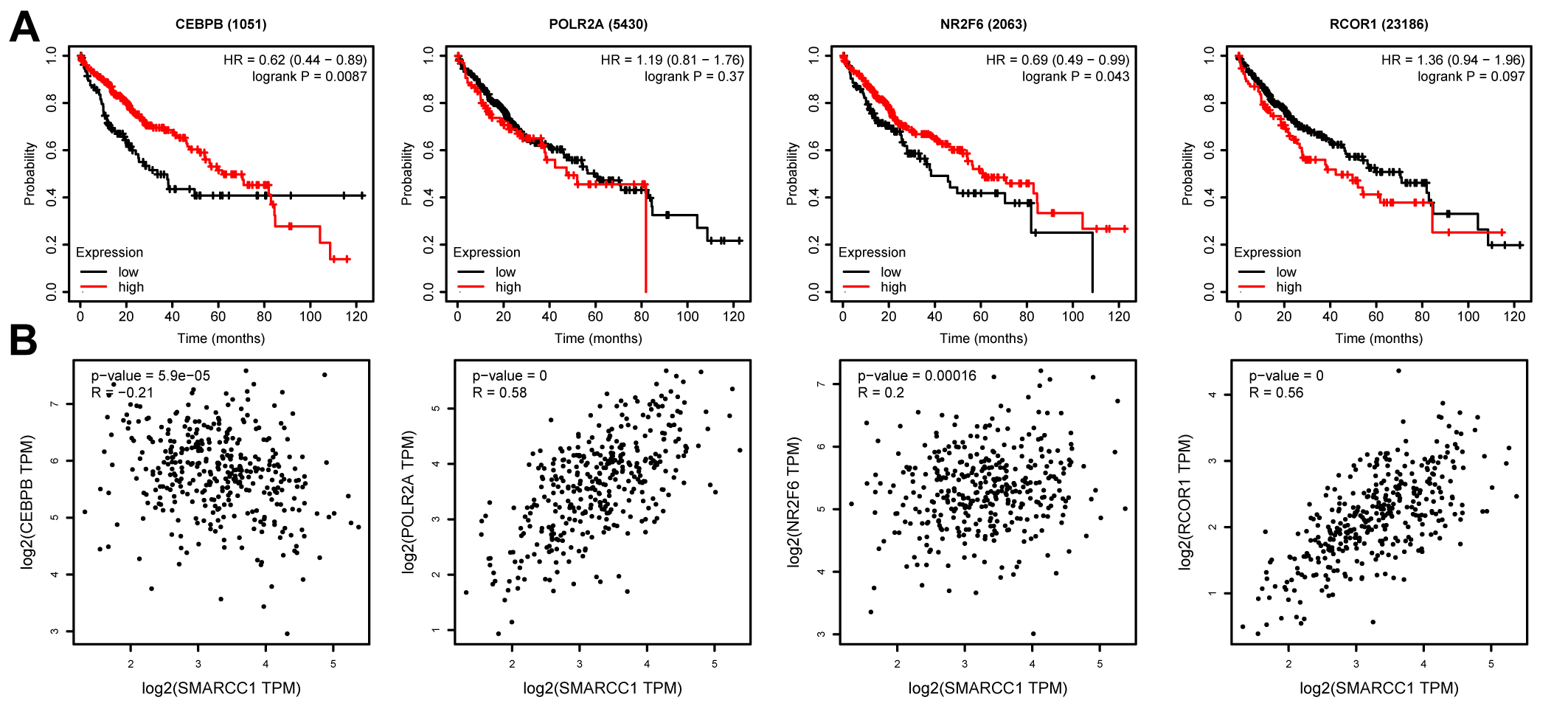

Supplement: Supplementary file 5 — Additional file 5: Figure 5. a The prognostic values of the other 4 transcription factors in HCC from Kaplan‐Meier Plotter online tool. b The relevance of SMARCC1 expression in relation to the other 4 transcription factors expression in HCC from GEPIA database. [file 12935_2021_2413_MOESM5_ESM.tif]

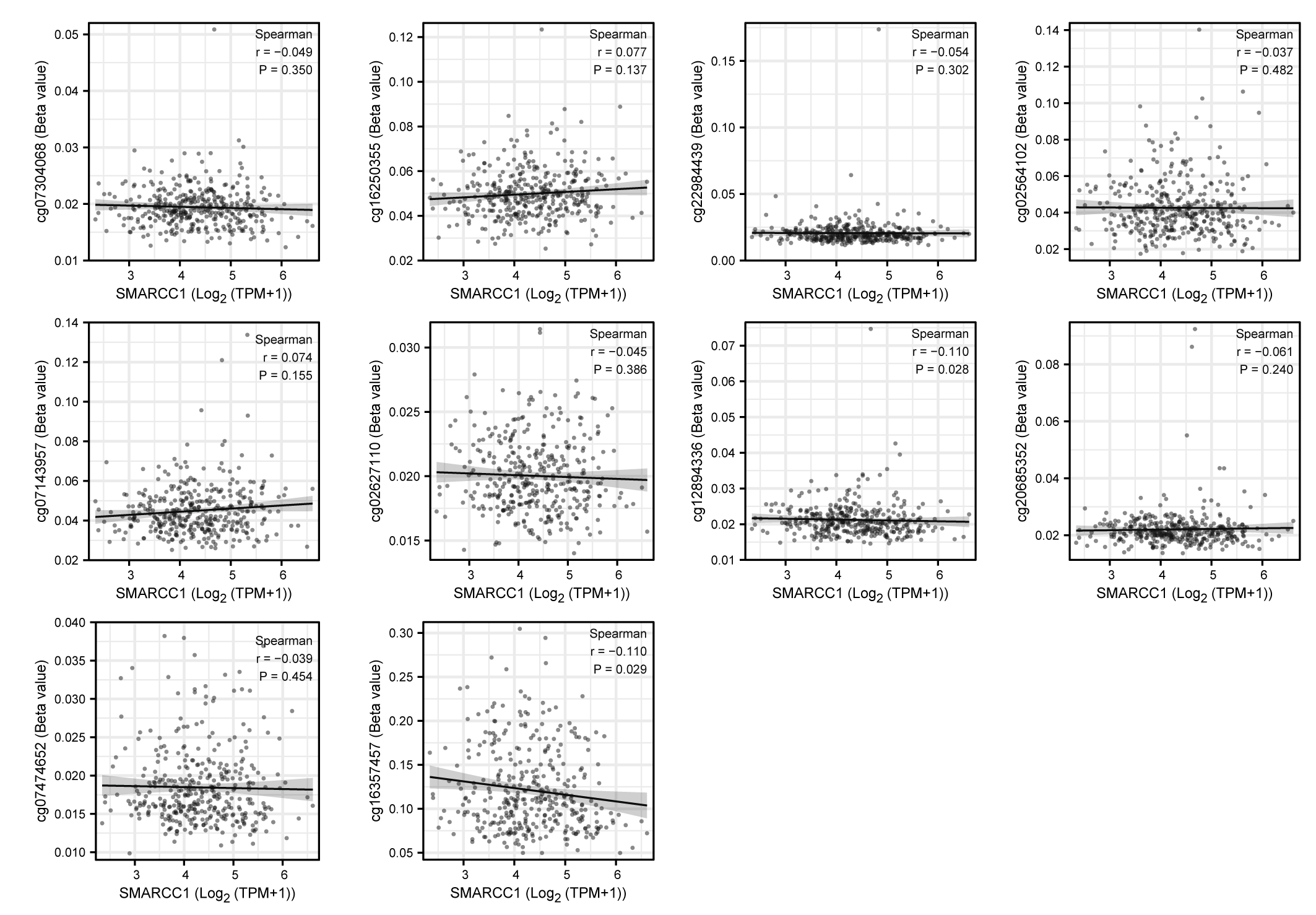

Supplement: Supplementary file 6 — Additional file 6: Figure 6. Relationships of SMARCC1 with the other 10 DNA methylation sites in HCC based on TCGA database. [file 12935_2021_2413_MOESM6_ESM.tif]
